# Supplementary material for: New Therapeutic Strategies in Retinal Vascular Diseases: A Lipid Target, Phosphatidylserine, and Annexin A5—A Future Theranostic Pairing in Ophthalmology
Source: Pharmaceuticals (Basel). 2024 Jul 24;17(8):979. doi: 10.3390/ph17080979 (PMC11357257; doi:10.3390/ph17080979)
Supplement: Supplementary file 1 [file pharmaceuticals-17-00979-s001.zip › pharmaceuticals-3055839-supplementary.pdf]

Expected effects	ANXV	Anti-VEGF
Impact on RBC adhesion	+	-
Anti-inflammatory	+	-
Anti-apoptotic	+	-
Repairs cell membranes	+	-
Improves circulation	Indirectly	-
Limits retinal area of retinal non perfusion	Indirectly	Uncertain
Reduces pathologic blood vessel formation	+	+
Reduces retinal swelling	Indirectly	+

Table S1. The expected clinical effects (based on the Mode of Action) for recombinant human Annexin A5 (ANXV) as compared to the current standard-of-care (anti-VEGF) in patients with Retinal Vein Occlusion.
